# *Drosophila* Model for Gut-Mediated Horizontal Transfer of Narrow- and Broad-Host-Range Plasmids

**DOI:** 10.1128/mSphere.00698-21

**Published:** 2021-10-20

**Authors:** Logan C. Ott, Mark Engelken, Sara M. Scott, Elizabeth M. McNeill, Melha Mellata

**Affiliations:** a Department of Food Science and Human Nutrition, Iowa State University, Ames, Iowa, USA; b Interdepartmental Microbiology Graduate Program, Iowa State University, Ames, Iowa, USA; University of Michigan—Ann Arbor

**Keywords:** *Drosophila*, horizontal gene transfer, plasmid incompatibility, sexual dimorphism

## Abstract

Horizontal gene transfer (HGT) is a driving force of microbial evolution. The gut of animals acts as a potent reservoir for the lateral transfer of virulence, fitness, and antimicrobial resistance genes through plasmids. Reduced-complexity models for the examination of host-microbe interactions involved in plasmid transfer are greatly desired. Thus, this study identifies the use of Drosophila melanogaster as a model organism for the conjugation of plasmids of various incompatibility groups in the gut. *Enterobacteriaceae* conjugation pairs were identified *in vitro* and used for oral inoculation of the *Drosophila* gut. Flies were enumerated for the donor, recipient, and transconjugant populations. Each donor-recipient pair was observed to persist in fly guts for the duration of the experiment. Gut concentrations of the donors and recipients were significantly different between male and female flies, with females generally demonstrating increased concentrations. Furthermore, host genetics significantly altered the concentrations of donors and recipients. However, transconjugant concentrations were not affected by host sex or genetics and were detected only in the IncPε and IncI1 plasmid groups. This study demonstrates Drosophila melanogaster as a model for gut-mediated plasmid transfer.

**IMPORTANCE** Microbial evolution in the gut of animals due to horizontal gene transfer (HGT) is of significant interest for microbial evolution as well as within the context of human and animal health. Microbial populations evolve within the host, and factors from the bacteria and host interact to regulate this evolution. However, little is currently known about how host and bacterial factors regulate plasmid-mediated HGT in the gut. This study demonstrates the use of *Drosophila* and the roles of sexual dimorphism as well as plasmid incompatibility groups in HGT in the gut.

## INTRODUCTION

The emergence of antibiotic-resistant bacteria (ARB) is a threat to agricultural and human health and safety (1–4). Antibiotic resistance (AR) genes (ARGs) emerge in numerous environments, including food processing, agriculture, waste treatment, and clinical environments (4–7). They can spread through horizontal gene transfer (HGT) between bacterial populations, resulting in the emergence of new strains with various combinations of AR, virulence, and metabolic genes (1, 8–20). HGT enables the rapid expansion of novel ARB strains in naive populations. The emergence and expansion of clinically relevant ARB strains can occur from two events, random mutation and selection by the low-dose use of antibiotics used in both health care and agriculture, or from the introduction of whole genes on mobile genetic elements into a naive bacterial host through transformation, transduction, or conjugation. The latter mobile pathway, conjugation, is currently believed to be the most significant in the emergence of novel ARB strains (21–23).

The conjugation process leading to the exchange of small to large plasmids or integrative conjugative elements (ICE) from one bacterial cell to another is prevalent in every environment where bacterial communities persist. The gut of animals is a common area of focus for studying microbial communities and has been indicated as a potential habitat for subsequent plasmid-mediated conjugation and HGT (12, 24–28). Few host-derived factors have previously been experimentally demonstrated to regulate the efficiency of conjugation between bacteria in the gut. Recently, host gut inflammation was shown to be a possible regulator of bacterial conjugation in a transgenic T cell-mediated inflammation model (28). Furthermore, a recent study from our laboratory has shown that a host’s genetics can also significantly impact plasmid-mediated HGT in the gut of a defined microbiota murine model (29).

Drosophila melanogaster is an arthropod used as a model for study in a multitude of scientific fields, from biopharmaceuticals to behavior and cellular development (30–34). These flies are useful due to their ease of propagation, maintenance, and genetic and egg manipulation and their reduced biological complexity (31, 33). While many bacterium-host interactions in *Drosophila* have been characterized outside HGT, there are still significant holes in the current knowledge regarding bacterial evolution in the gut. The roles that host and bacterial factors play in the regulation of HGT on the surfaces of epithelial layers, both skin and gut, have not been well studied.

*Drosophila* flies, as with all arthropods, lack adaptive immunity like those found in mammals such as mice and humans (35). As such, they rely to a greater extent on mechanisms of innate immunity for the modulation and control of the resident gut microbiota, pathobionts, and pathogens. Due to this limited repertoire of immune function, *Drosophila* and other arthropods often act as potent vectors for the spread of microbial constituents between environments through mechanical and biological vectoring (36). Although most bacteria colonize the gut of *Drosophila* only transiently, temporary colonization may be significant enough for HGT to occur in the gut and result in dissemination.

Importantly, fruit flies (*Drosophila*) act as laboratory models for other significant arthropod hosts such as mosquitos (*Culicoidea*) and houseflies (*Musca*). For many of these arthropod pests, a primary food source is the fecal waste of agricultural animals in natural and built environments. Similarly, the fruit fly diet consists of the microbes and nutrients found on the surfaces of decomposing fruit, including yeast, molds, as well as dietary and contaminating bacteria. These fruit waste and decomposition sources occur in complex agricultural environments that have been demonstrated to be potent reservoirs of antimicrobial resistance and pathogens (37). Food preferences and the ability to cover large areas through flight implicate these hosts as vectors for transferring antimicrobial resistance and pathogens from these high-risk environments through the broader environment, facilitating rapid microbial evolution upon introduction to naive populations.

Understanding the complex bacterium-host interactions that are involved in the regulation of bacterial plasmid-mediated HGT is important for understanding the basic mechanism of HGT, the emergence of ARB in the environment, as well as the significance of these events in the global crisis of ARG emergence. This study aims to determine the suitability of Drosophila melanogaster as a model for and biological factors regulating HGT between plasmids of differing incompatibility types within the gut.

## RESULTS

### Bacterial conjugation pairs produce functional transconjugants after incubation under *in vitro Drosophila* conditions.

**FIG 1:**
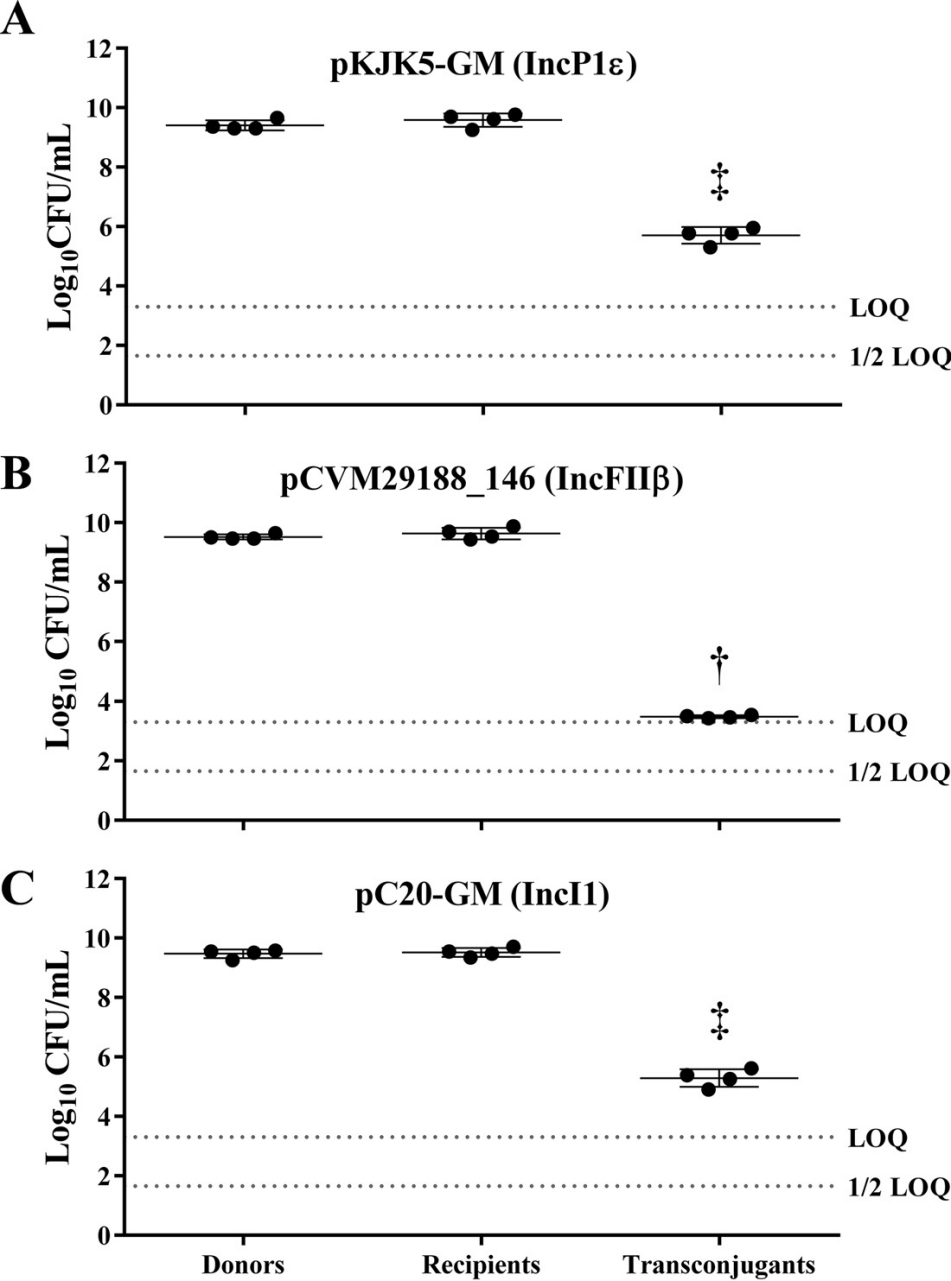
*In vitro* broth conjugation. Enumeration of donor (left), recipient (middle), and transconjugant (right) strains in liquid broth was performed. Conjugation was done between donor strain SP915 harboring plasmid pKJK5-GM (A), pCVM29188_146 (B), or pC20-GM (C) and recipient strain HS-4. Bars represent the means from four individual replicates, and error bars represent standard deviations above and below the means. The horizontal dashed line is the limit of quantification (LOQ). Symbols above the bars indicate the significance group. Bars with alternate significance symbols are significantly different (*P ≤ *0.05).

To determine if each bacterial conjugation pair produces functional transconjugants under *in vitro* conditions mimicking the fly gut, liquid broth conjugations between selected donor-recipient pairs were conducted. Conjugations were conducted in a *Drosophila* incubator at 25°C for 5 h during the light phase of fly culture (Fig. 1). Independent bacterial conjugations between the donor Escherichia coli SP915 harboring pKJK5-GM (MM0001) (Fig. 1A), pCVM29188_146 (MM0002) (Fig. 1B), or pC20-GM (MM0003) (Fig. 1C) and the plasmidless recipient E. coli HS-4 strain resulted in detectable transconjugants in every replicate. Conjugation between E. coli MM0001, MM0002, or MM0003 and HS-4 resulted in mean conjugation frequencies of 2.15 × 10^−4^ ± 8.71 × 10^−5^, 9.58 × 10^−7^ ± 2.61 × 10^−7^, and 6.93 × 10^−5^ ± 2.76 × 10^−5^ transconjugants per donor, respectively (Fig. 1). Transconjugants were detected at significantly lower levels in MM0002 conjugations.

### Donor and recipient strains are detectable in the gut of Drosophila melanogaster *in vivo*.

**FIG 2:**
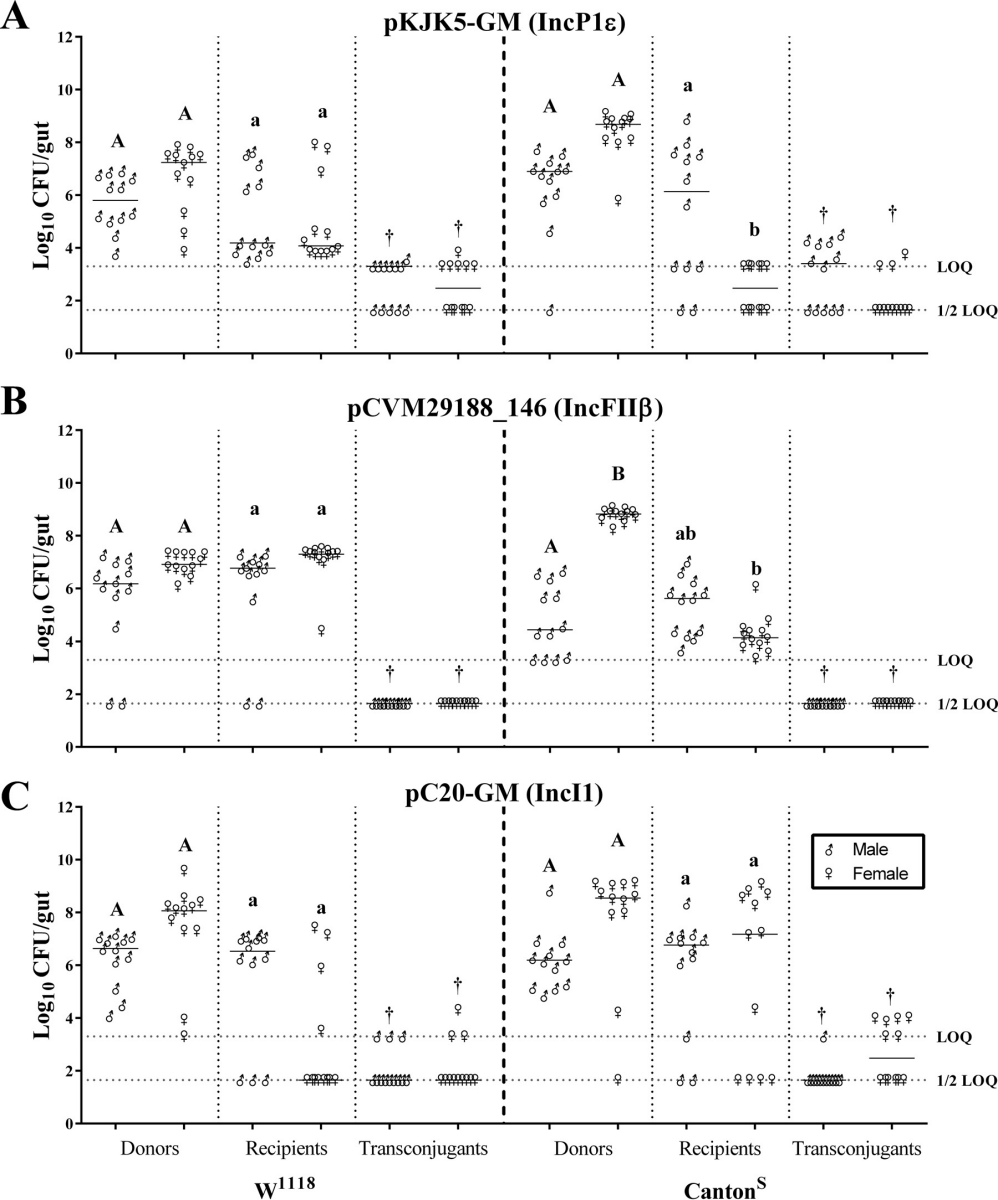
*In vivo* inoculation and conjugation in adult Drosophila melanogaster. Enumeration of donors, recipients, and transconjugants in male and female guts of W^1118^ (left) and Canton^S^ (right) flies was performed. Each marker represents an individual fly replicate (*n* = 12/group), and each bar represents the median. *P* values of ≤0.05 were considered significant. Groups with different letters were significantly different. Significance was determined individually between host sex and genetics with respect to each population by nonparametric Kruskal-Wallis one-way ANOVA. The upper horizontal dashed line is the limit of quantification (LOQ), and the lower dashed line is the 1/2 point between zero and the LOQ. Samples that tested negative were assigned a value at the half-LOQ line, and undiluted samples with greater than 0 but fewer than 20 colonies were assigned a value at the LOQ line.

Each donor-recipient pair was assayed for the ability to persist in the gut of flies for 1 h following inoculation with recipients. Donor and recipient strains were identified in the gut of *Drosophila* of either the W^1118^ or Canton^S^ fly strain for all three conjugation pairs tested (Fig. 2). The conjugation pair of MM0001 colonized the guts of male and female W^1118^ flies at 5.72 ± 1.04 and 6.60 ± 1.32 CFU/gut, respectively (Fig. 2A). The recipient HS-4 strain colonized the gut at a reduced concentration compared to that in males; however, the difference was not statistically significant. The HS-4 recipient colonized male and female guts at means of 5.20 ± 1.64 and 4.90 ± 1.61 CFU/gut, respectively. The MM0001 donor and HS-4 recipient pair colonized the guts of the Canton^S^ wild-type strain, with the donor strain being detected in male and female guts at 6.26 ± 1.68 and 8.34 ± 0.89 CFU/gut, respectively. However, in Canton^S^ flies, one male fly demonstrated populations below the theoretical limit of quantification (LOQ). Likewise, the HS-4 recipient colonized the guts of male and female Canton^S^ flies at 5.41 ± 2.61 and 2.48 ± 0.86 CFU/gut, respectively.

Inoculation of the MM0002 donor and HS-4 recipient conjugation pair resulted in detectable populations in both male and female W^1118^ and Canton^S^ flies (Fig. 2B). The donor strain MM0002 was detected in the guts of male and female W^1118^ and Canton^S^ flies at 5.54 ± 1.96, 6.90 ± 0.41, 4.79 ± 1.35, and 8.75 ± 0.23 CFU/gut, respectively. MM0002 donors were not detected in two male W^1118^ flies and were detected at levels below the theoretical LOQ in three male Canton^S^ flies. HS-4 was detected in the guts of male and female W^1118^ and Canton^S^ flies at 5.94 ± 2.05, 7.05 ± 0.85, 5.30 ± 1.10, and 4.22 ± 0.70 CFU/gut, respectively. MM0002 donors were undetectable in two male W^1118^ flies and below detectable limits in one female Canton^S^ fly.

Inoculation of the MM0003 donor and HS-4 recipient conjugation pair resulted in detectable populations in both male and female flies of either the W^1118^ or Canton^S^ fly strain (Fig. 2C). Donors were detected in the guts of male and female W^1118^ and Canton^S^ flies at means of 6.22 ± 1.07, 7.38 ± 1.86, 6.17 ± 1.09, and 7.68 ± 2.32 CFU/gut, respectively. MM0003 donors were observed below the LOQ in one female W^1118^ fly and two female Canton^S^ flies. HS-4 was detected in the guts of male and female W^1118^ and Canton^S^ flies at 5.47 ± 2.33, 3.10 ± 2.33, 5.77 ± 2.26, and 5.72 ± 3.25 CFU/gut, respectively. HS-4 recipients were undetectable in three male and seven female W^1118^ flies, below detectable limits in one male Canton^S^ fly, and undetectable in two male and four female Canton^S^ flies.

### Host sex affects donor and recipient abundances in the gut.

To determine if host sex affects abundance and conjugation, males and females were segregated and tested in independent groups in *in vivo* assays. The MM0002 donor colonized the guts of female Canton^S^ flies significantly more than the guts of males of the same genetic background (Fig. 2B). This trend was observed in both W^1118^ and Canton^S^ flies for all other donor and genotype combinations; however, the differences were not statistically significant. No significant difference was observed between males and females of the MM0003 donor groups (Fig. 2C). Furthermore, the recipient HS-4 strain colonized the guts of male Canton^S^ flies at significantly higher concentrations than those of female Canton^S^ flies (Fig. 2A and B). However, this trend was not consistent across fly genotypes and plasmid groups.

### Conjugation of the broad-host-range IncP and narrow-host-range IncI plasmids occurs in the gut of Drosophila melanogaster.

The presence and enumeration of transconjugant populations in the guts of inoculated flies were determined. In inoculations with the MM0001 donor and HS-4 recipient, transconjugants were detected in a subset of both male and female flies of either the W^1118^ or Canton^S^ genetic background. Specifically, seven male and six female W^1118^ flies demonstrated transconjugant populations, of which one fly in each group had populations detected at levels higher than the theoretical LOQ. Seven males and three female Canton^S^ flies demonstrated detectable transconjugants, with six male flies and one female fly demonstrating transconjugant populations at levels higher than the theoretical LOQ.

In inoculations with the MM0002 donor and HS-4 recipient, no transconjugants were detected in either male or female W^1118^ flies. Furthermore, no transconjugants were detected in either male or female Canton^S^ flies. Postenumeration enrichment of fly homogenates in transconjugant-selective Luria-Bertani (LB) medium yielded no detectable transconjugants, indicating their absence in fly homogenates.

In inoculations with the MM0003 donor and HS-4 recipient, transconjugants were detected in a portion of both male and female flies of either the W^1118^ or Canton^S^ genetic background. Specifically, three male and female W^1118^ flies demonstrated transconjugant populations, of which one female fly demonstrated a population at a level higher than the theoretical LOQ. One male and six female Canton^S^ flies demonstrated detectable transconjugants, with four female flies demonstrating transconjugant populations at levels higher than the theoretical LOQ.

### Genetic background affects abundance but not conjugation in the gut.

To determine if host genetics affect the abundance and resulting conjugation in the gut, both W^1118^ and Canton^S^ flies were assayed for bacterial enumeration and conjugation efficiency following oral inoculation with donor-recipient pairs (Fig. 2). Female Canton^S^ flies were colonized by recipient HS-4 at significantly lower (*P < *0.05) concentrations than those of the recipient in female W^1118^ flies when inoculated with SP915 harboring either pKJK5-GM or pCVM29188_146 (Fig. 2A and B). The populations of transconjugants were not significantly different between genetic backgrounds in groups where conjugation was detected (pKJK5-GM and pC20-GM) (Fig. 2A and B).

## DISCUSSION

Few studies have identified specific factors that may be important in regulating bacterium-to-bacterium plasmid conjugation in the gut. These limited factors include host genetics and inflammation (28, 29). Much is still unknown about the interactions of the host and bacteria in this dynamic and complex environment as it relates to the evolution of bacterial populations through the direct transfer of DNA from one strain to another. As such, a model for the study of conjugation in a simplified and controlled host environment is desired. Mice have previously presented a limited-use model for this case, as the advent of altered Schadler flora (ASF) mice provided control over the diet, environment, genetics, microbiota, and other important host factors. Murine models still present great variability and limitations due to their natural complexity (28, 29).

While a few studies of bacterial conjugation have been completed in the common housefly (Musca domestica), no studies have yet used the laboratory research model Drosophila melanogaster as a host for gut-mediated HGT (38, 39). *Drosophila* has historically been used as a model organism to better understand human and animal physiology and development as well as a model for pest arthropods involved in human and animal health and agricultural productivity. *Drosophila* presents as an interesting model to study HGT due to its many benefits. These include the cost of use, generation time, genetic toolbox, as well as simplified host immunity. *Drosophila* flies maintain moderately complex innate immunity; however, they lack an adaptive immune system, resulting in a reduced number of variables associated with host-bacterium interactions mediated by the immune system. Additionally, the limited diversity and abundance of the natural gut microbiota decrease gut complexity, as most microbes in the gut of *Drosophila* are transient and are generally limited compared to those of other animal hosts. One of the confounding factors that prevent the productive study of *Enterobacteriaceae* in mouse guts is competitive exclusion by the gut microbiota (28, 29). Both characteristics make *Drosophila* a desirable model for the study of the complex interactions that are proposed to be involved in regulating HGT in the gut.

The use of *Drosophila* for HGT studies may also allow the study of behavioral and biological differences that contribute to sexual dimorphisms as well as their role in the process of colonization and bacterial conjugation. Links have recently been established between genetic sexual identifications and differences in the immune responses to infection of males and females across the animal kingdom (38, 40, 41). This supports other research that has previously shown that differences in infection susceptibility of *Drosophila* can be distinct for males and females and even more so after mating (40, 41). Schwenke and Lazzaro described the role of juvenile hormone in suppressing the immune response in mated female Drosophila melanogaster flies after systemic infection (40). In the present study, we showed increased abundances of donor and recipient strains within the guts of mated female flies, which may be a consistent response due to the differences in the immune functions of male and female flies or behavioral differences between males and females in the consumption of food. Future studies are needed to determine causality.

In the gut of *Drosophila*, innate immunity is primarily mediated by the physical barriers of the peritrophic matrix and mucus layers. Additionally, bacterially derived uracil and peptidoglycan induce the dual-oxidase (DUOX) pathway, or the Imd pathway, with the subsequent activation of both antimicrobial peptide secretion as well as the DUOX pathway (35). Conversely, systemic immune responses are mediated by peptidoglycan recognition and the activation of Imd in addition to the recognition of microbe-associated molecular patterns (MAMPs) through intra- and extracellular peptidoglycan recognition proteins (PGRPs). Previous studies have demonstrated distinct sexual dimorphisms between males and females in both the Imd and Toll-mediated systemic responses (42–44). In both cases, mated female flies are significantly more susceptible to bacterial pathogens during systemic infections by Gram-positive and Gram-negative bacteria in addition to fungal pathogens (42, 43, 45). The role of host factors conferred by mating status and sex in systemic infections established in the literature may, or may not, fully extend to gut abundance and the productive transfer of plasmids. Toll receptors are not located on enterocytes in the gut, as they are on hemocytes and other host cells, and it is not yet clear if the Imd pathway alone is sufficient to explain the sexual dimorphisms observed in our study. Further experiments to determine the roles of this immune regulator and sexual dimorphism are under way.

The further use of this model may help to understand better the role that sex plays in the process of conjugation as well as the role of sex differences in gastrointestinal abundance and bacterium-host interactions overall. Studies addressing these as-yet-unknown factors involved in controlling abundance between male and female host flies in oral inoculation and gut-mediated HGT are under way.

While the concentrations of bacteria in the gut are significantly different between male and mated female flies, the conjugation rates between donor and recipient flies are not observed to be. However, plasmid biology appears to be a potent regulator of conjugation within fly hosts. Previous studies from our laboratory and others have shown high levels of conjugation between *Enterobacteriaceae* involving the transfer of large antimicrobial resistance plasmids from the IncF and IncI groups (28, 29).

In this study, we tested plasmids from the broad-host-range incompatibility group P as well as plasmids from the narrow-host-range incompatibility groups F and I (6, 46, 47). These plasmids were housed within a common *Enterobacteriaceae* E. coli SP915 donor to eliminate donor strain effects. *In vitro* conjugation experiments using this donor and the plasmid-free E. coli HS-4 recipient demonstrated detectable transconjugants in each conjugation pair under conditions mimicking those in *Drosophila*. Additionally, conjugation frequencies between each conjugation pair in *in vitro* assays were not significantly different from each other. However, in *in vivo* experiments, we detected successful conjugation only from plasmid donors of the IncP and IncI incompatibility types even with significant abundances in flies with both donor and recipient strains in the IncF groups. IncP plasmids are broad-host-range plasmids with a surprisingly broad functional host range. Klümper et al. demonstrated the transferability of IncP plasmids in soil and wastewater microbial communities with interspecies, intergenus, and interphylum transfer, even if only transiently (6). The biology of plasmids varies greatly according to incompatibility types such as the relaxase family MOB (for mobility), iterons, *rep* and *cop* genes, RNA interference (RNAi) sequences, and replication origins (48). A detailed study of the genetic composition of the plasmids used in this study is under way as complete genome sequences of some plasmids used in this study are not readily available.

It is not yet clear what plasmid-encoded or -conferred factors are responsible for the fertility of IncP and IncI and the repression of IncF plasmid conjugation in the gut. However, it has previously been shown that the conjugation machinery encoded by plasmids of different incompatibility groups varies dramatically (49). IncP plasmids demonstrate robust conjugation in comparison to IncF and IncI plasmid types (50). Bates et al. demonstrated unusually robust conjugative transfer between E. coli and the eukaryotic fungal species Saccharomyces cerevisiae (50) where increased conjugation was determined to be mediated by the Tra2 core and the mating pair formation (MPF) proteins associated with IncP plasmids. Furthermore, the IncP plasmid used in this study is smaller than the IncF or IncI plasmid used. It is not clear how this difference in plasmid material length may affect conjugation in the complex gut environment where bacterial cells are mainly transient and not static, as is the case for *in vitro* conjugation.

Conjugation occurred on the surface of fly medium in the absence of flies in all plasmid groups, indicating the possibility of conjugation in the environment; however, no transconjugants were detected in either the recipient inoculated or final fly medium tubes, indicating that conjugation was occurring in the flies themselves.

Finally, this study examined conjugation in the guts of flies of two genetic backgrounds. We identified previously that host genetics may play a significant role in the regulation of bacterial conjugation in the gut (29). Host genetics was confirmed to affect colonization of the gut by donor and recipient strains; however, no effect was identified regarding conjugation. This result may be due to the variability of the observed conjugation that may be masking true trends or differences between males and females or even host genetics. Future studies with increased sample sizes will be required to further identify if host genetics or sex affects the presence and rate of conjugation in the gut. Additionally, previous literature identified large variation in immune responses in *Drosophila* as a function of host genetics, supporting the need to examine HGT and bacterial abundance in various genetic backgrounds (51). We are currently testing the role of host genetics in the productive transfer of plasmids in the gut of *Drosophila* in two genetic models; however, additional studies with a larger number of genetic strains are required to elucidate further the role of host genetics in regulating bacterial conjugation in the gut of *Drosophila.*

## MATERIALS AND METHODS

### Bacterial strains and plasmids.

Escherichia coli strain HS-4 Rif^r^ was used as the plasmidless recipient strain in all *in vitro* and *in vivo* conjugation assays. To control for donor strain variation, the modified laboratory E. coli K-12 strain MG1655::*lacI*^q^-Plpp-*mCherry* (SP915) was used as a common donor for the plasmids pKJK5-GM, pCVM29188_146, and pC20-GM, referred to as MM0001, MM0002, and MM0003, respectively, throughout (Table 1). Briefly, initial plasmid donor strains were conjugated with SP195 in *in vitro* broth conjugations as described below. The resulting transconjugants were verified by plasmid gel electrophoresis and stored in glycerol at −80°C for later use. Prior to each experiment, bacterial cultures were freshly streaked from −80°C glycerol stocks and incubated on Luria-Bertani (LB) plates supplemented with antibiotics selecting for chromosomal and plasmid resistance. Cultures were incubated overnight at 37°C prior to each assay, and purity was confirmed by the lack of resistance to alternate resistance markers.

**TABLE 1:** Bacterial strains and plasmids^*a*^

Strain or plasmid	Role	Plasmid	Relevant property(ies)	Reference
Strains				
Salmonella enterica serovar Kentucky				
CVM29188	Donor	pCVM29188_146	Commercial chicken breast isolate	46
		pCVM29188_101		
		pCVM29188_46		
E. coli				
SP915	Recipient		K-12 MG1655 lab strain; Km^r^	6
SP961	Donor	pKJK5-GM	K-12 DH10B lab strain; Km^r^	6
SP1414	Donor	pC20-GM	Clinical urinary tract isolate; Km^r^	47
HS-4	Recipient		Human commensal isolate; spontaneous rifampicin resistance	29
MM0001	Donor	pKJK5-GM	SP915 transconjugant	This study
MM0002	Donor	pCVM29188_146	SP915 transconjugant	This study
MM0003	Donor	pC20-GM	SP915 transconjugant	This study

Plasmids				
Narrow host range				
pCVM29188_146			IncFIB; Tet^r^ Str^r^	46
pCVM29188_101			IncI1; Ctx^r^	46
pCVM29188_46			IncFII	46
pC20-GM			IncI [PA10403-gfpmut3]; Ctx^r^	47
Broad host range				
pKJK5-GM			IncP-1ε [PA10403-gfpmut3]; Tet^r^	6

a Abbreviations: Km^r^, kanamycin resistant; Rif^r^, rifampicin resistant; Ctx^r^, cefotaxime resistant.

### *In vitro* conjugation assays.

The ability of the donor strains to transfer AR plasmids was examined using a liquid culture mating technique as previously described (29). Initial mating pairs included (i) MM0001 and HS-4, (ii) SP915(pCVM2918_146) and HS-4, and (iii) MM0003 and HS-4. Briefly, strains were grown in 5 ml of LB broth with the appropriate antibiotics overnight at 37°C with shaking at 200 rpm. On the next day, the cultures were adjusted to an optical density at 600 nm (OD_600_) of ∼1.0, and 1 ml of the culture was centrifuged at 16,000 × *g* for 5 min and resuspended in 0.5 ml of LB broth. The donor and recipient strains were mixed at a 1:1 ratio in triplicate and incubated for 6 h at 25°C. Dilutions of the mating mixtures were plated onto MacConkey agar plates containing antibiotics selecting for the recipients and transconjugants, donors and transconjugants, or transconjugants only. The antibiotics used per conjugation are described in Table 1. Antibiotics were purchased from Fisher Scientific or Sigma-Aldrich. The transconjugant frequency was calculated and reported as the total population of transconjugant CFU divided by the total population of donor CFU for each replicate.

### Plasmid profiling and typing.

Plasmid profiling was conducted as described previously (29, 52). Briefly, transconjugant isolates were grown in LB broth to an OD_600_ of ∼0.8. Cultures were then centrifuged and resuspended in 200 μl resuspension buffer (0.04 M Tris-acetate [pH 8.0], 2 mM EDTA). Next, 400 μl of lysis buffer (0.05 M Tris, 3% SDS [pH 12.5]) was added, and cultures were mixed by inversion. Cultures were incubated at room temperature for 60 min. Following incubation, 600 μl of phenol-chloroform (1:1) was added, and cultures were mixed gently by inversion. Phases were then separated by centrifugation at 10,000 × *g* for 10 min at room temperature. The top aqueous solution was transferred to a new tube and used directly for 0.5% agarose gel electrophoresis as previously described (52).

### Fly strains and rearing.

The genetic background W^1118^ and wild-type Canton^S^ fly lines were maintained at 25°C on Jazz mix *Drosophila* medium (Fisher, Waltham, MA, USA) prepared according to the manufacturer’s instructions. Jazz mix medium consists of brown sugar, cornmeal, yeast, agar, benzoic acid, methylparaben, and propionic acid; however, specific concentrations of each component are not available. Prior to conjugation assays, flies were transferred to defined Bloomington *Drosophila* Stock Center (BDSC) cornmeal medium prepared without the addition of propionate (53). Bacterial strains used in this study were confirmed to grow on fresh BDSC cornmeal fly medium, and conjugation occurs on fly medium surfaces in the absence of flies. Fly incubations were conducted in a refrigerated incubator at 25°C under 12-h/12-h light/dark cycles and 70% humidity.

### Fly inoculation and bacterial enumeration.

**FIG 3:**
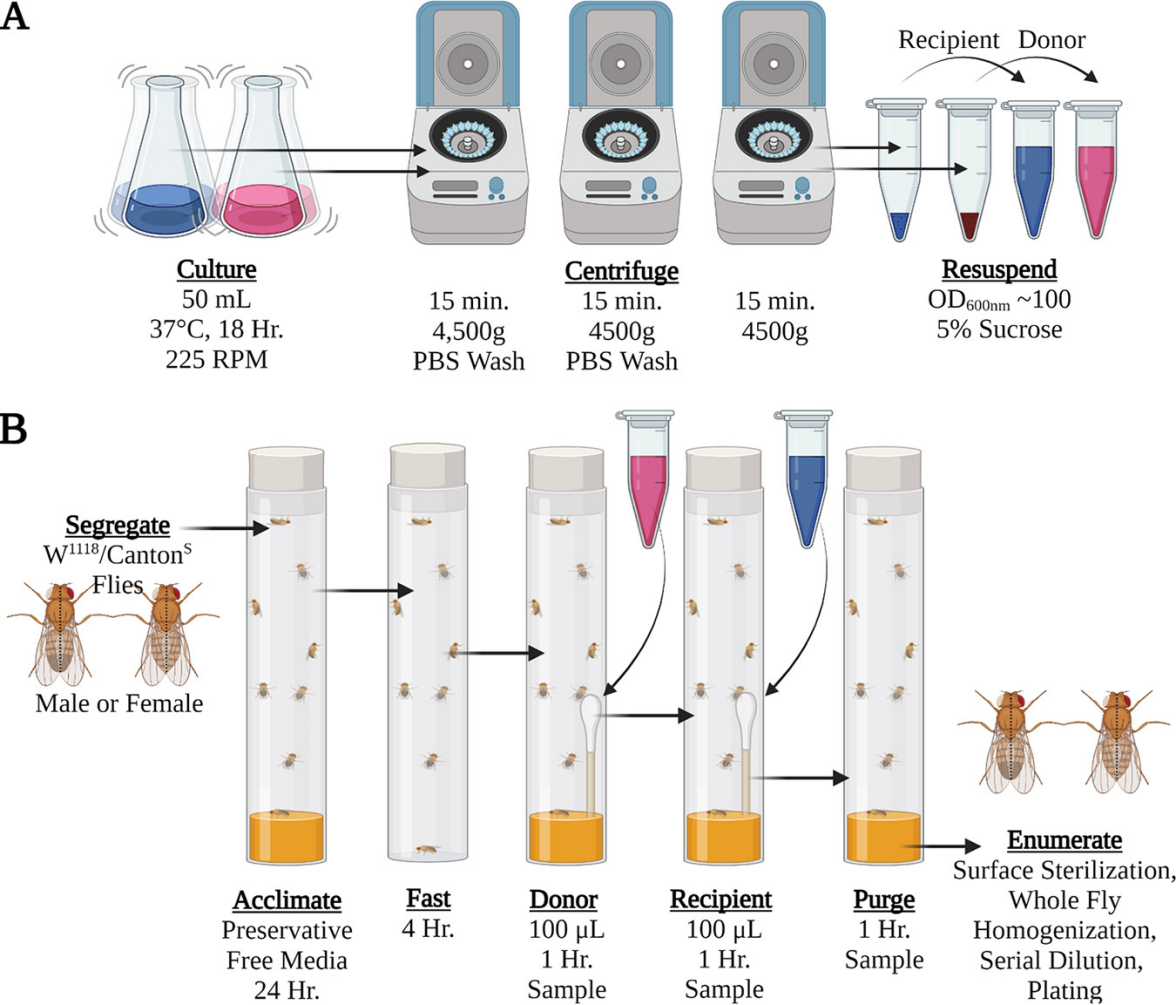
Bacterial culture and fly inoculation methods. (A) Bacterial cultures were prepared from fresh shaking cultures grown overnight and standardized to an OD_600_ of 100 in 5% sterile sucrose in ddH_2_O. (B) Donor (pink) and recipient (blue) suspensions were used for oral colonization in segregated male and female W^1118^ or Canton^S^ flies. Flies were acclimated, fasted, orally fed donor and then recipient populations, and then placed into fresh tubes for an hour prior to surface sterilization, homogenization, and enumeration.

Fresh cultures of donor and recipient strains (Table 1) grown overnight were used to inoculate 50 ml of LB broth in Erlenmeyer flasks by selecting 1 to 2 isolated colonies and suspending them in LB broth supplemented with the appropriate antibiotics (Fig. 3A). Cultures were incubated for 18 h or until the OD_600_ reached >1.0. The requisite volume of the culture grown overnight was pelleted at 4,500 × *g* for 15 min. Pellets were washed with phosphate-buffered saline (PBS) twice before suspension in 5% sucrose in sterile double-distilled water (ddH_2_O) to a final OD_600_ of approximately 100. For conjugation experiments, flies were fasted in clean empty fly tubes for a period of 4 h in groups of males or females (*n* = 12/group) 5 to 7 days after eclosion (Fig. 3B). A 100-μl volume of the donor cell suspension was applied to the tip of a sterile cotton swab inserted into fresh BDSC cornmeal medium in a new tube. Flies were transferred into the tube and allowed access to fly food and the wetted cotton swab *ad libitum* for 1 h. Recipient cell suspensions (100 μl) were likewise used to wet the cotton end of a new sterile swab and placed into a fresh BDSC fly medium tube. Flies were transferred to the recipient-inoculated fly tube and incubated for 1 h. Following incubation, flies were transferred to fresh BDSC fly medium tubes and incubated for one additional hour. All incubations occurred under standard *Drosophila* conditions as described above.

After the final incubation period, flies were CO_2_ anesthetized, transferred individually into sterile microcentrifuge tubes, surface sterilized in 250 μl of 70% ethanol for 90 s, rinsed with sterile 1× PBS for 90 s, and then individually transferred to microcentrifuge tubes containing 100 μl of fresh sterile PBS. Bacterial populations of the gut were inferred from whole-fly bacterial enumeration following ethanol surface sterilization (54). Individual surface-sterilized flies were homogenized by micropestle homogenization. Briefly, sterile plastic micropestles (Pellet Pestles; Fisher, Waltham, MA) were used to grind and homogenize fly bodies for a minimum of 90 s or until all fly parts were no longer visible. Fly homogenates were then serially diluted in sterile PBS in five 10-fold dilutions and plated onto MacConkey agar supplemented with antibiotics targeting the donors and transconjugants, recipients and transconjugants, or only transconjugant populations as described above. The CFU of each population were back-calculated per fly gut, donor and recipient populations were reported as CFU per gut, transconjugants were reported as CFU per gut, and the conjugation frequency was calculated as described above for *in vitro* assays.

### Statistical analysis.

Statistical analysis was completed using the GraphPad Prism 6 software suite. Separate comparisons between sex (male and female) and genotype (W^1118^ and Canton^S^) were completed. To compare the mean ranks between groups, nonparametric Kruskal-Wallis analysis of variance (ANOVA) was used, and *post hoc* Dunn’s correction for multiple comparisons was applied. *P* values of ≤0.05 were considered significant.
